# *In vitro* analysis of antiviral immune response against avian influenza virus in chicken tracheal epithelial cells

**DOI:** 10.5713/ab.24.0117

**Published:** 2024-08-22

**Authors:** Jubi Heo, Thi Hao Vu, CH Kim, Anh Duc Truong, Yeong Ho Hong

**Affiliations:** 1 Department of Animal Science and Technology, Chung-Ang University, Anseong 17546, Korea; 2 Department of Biochemistry and Immunology, National Institute of Veterinary Research, Dong Da, Hanoi 100000, Vietnam

**Keywords:** Avian Influenza Virus, H5N1, Immune Response, Junction Complex, Signaling Pathway, Tracheal Epithelial Cell

## Abstract

**Objective:**

Avian influenza virus (AIV) infections first affect the respiratory tract of chickens. The epithelial cells activate the host immune system, which leads to the induction of immune-related genes and the production of antiviral molecules against external environmental pathogens. In this study, we used chicken tracheal epithelial cells (TECs) *in vitro* model to investigate the immune response of the chicken respiratory tract against avian respiratory virus infections.

**Methods:**

Eighteen-day-old embryonic chicken eggs were used to culture the primary chicken TECs. Reverse transcription-polymerase chain reaction (RT-PCR) and immunocytochemistry (ICC) analysis of epithelial cell-specific gene makers were performed to confirm the characteristics, morphology, and growth pattern of primary cultured chicken TECs. Moreover, to investigate the cellular immune response to AIV infection or polyinosinic-polycytidylic acid (poly [I:C]) treatment, the TECs were infected with the H5N1 virus or poly (I:C). Then, immune responses were validated by RT-qPCR and western blotting.

**Results:**

The TECs exhibited polygonal morphology and formed colony-type cell clusters. The RT-qPCR results showed that H5N1 infection induced a significant expression of antiviral genes in TECs. We found that TECs treated with poly (I:C) and exposed to AIV infection-mediated activation of signaling pathways, leading to the production of antiviral molecules (e.g., pro-inflammatory cytokines and chemokines), were damaged due to the loss of junction proteins. We observed the activation of the nuclear factor kappa B and mitogen-activated protein kinase (MAPK) pathways, which are involved in inflammatory response by modulating the release of pro-inflammatory cytokines and chemokines in TECs treated with poly (I:C) and pathway inhibitors. Furthermore, our findings indicated that poly (I:C) treatment compromises the epithelial cell barrier by affecting junction proteins in the cell membrane.

**Conclusion:**

Our study highlights the utility of *in vitro* TEC models for unraveling the mechanisms of viral infection and understanding host immune responses in the chicken respiratory tract.

## INTRODUCTION

Viral respiratory infections in chickens affect poultry welfare and lead to economic losses in the poultry industry worldwide. Among them, avian influenza, which is a zoonotic disease caused by the influenza virus, infects various organisms, including humans. Avian influenza viruses belong to the *Orthomyxoviridae* family [1] and are categorized as low pathogenic avian influenza viruses (LPAIVs) and highly pathogenic avian influenza viruses (HPAIVs). The H5N1 subtype that originated in Hong Kong is transmissible to humans and has spread beyond Asia to the rest of the world [2]. Infection with this HPAIV causes severe symptoms and high mortality [1].

Hemagglutinin (HA) protein, a component of the AIV, which binds to the host cell surface receptor targets glycosylated oligosaccharides at the end of sialic acid (SA) residues that serve as viral receptors. In birds and mammals, influenza viruses mainly target and replicate in cells of the terminal portion of the lower respiratory tract (LRT), notably in the trachea and lungs. They achieve this by binding to the SA α2,3-galactose receptor on the epithelial cell surface. Viral replication in the tracheal region can result in infection of the terminal part of the LRT [3]. Host specificity and infectivity of AIV are influenced by mutations in the *HA* gene. The avian influenza H5N1 subtype primarily targets airway epithelial cells through the α2,3- and α2,6-type receptors [4]. Consequently, the H5N1 subtype causes severe disease because it can target and infect α2,6 receptors, similar to human and porcine influenza virus strains.

Tracheal epithelial cells (TECs) play a pivotal role in the defense against external pathogens, forming a physical barrier and regulating innate and adaptive immunity [5]. These cells, located in the upper respiratory tract, are targeted by both LPAIVs and HPAIVs, underscoring their significance in the host’s defense mechanisms [6]. The tracheal epithelium, crucial in initiating and amplifying host defense mechanisms against AIV, is actively involved in the immune response and inflammation of the respiratory system [7]. H5N1 HPAI viruses are identified by pattern-recognition receptors (PRRs) on the cell membrane, activating various immune-related pathways including the toll-like receptor (TLR), Janus kinase/signal transducers and activators of transcription (JAK/STAT), mitogen-activated protein kinase (MAPK), and nuclear factor kappa B (NF-κB) signaling pathways [8]. Therefore, TECs induce the production of host defense molecules, including antimicrobial, and antiviral proteins along with pro-inflammatory cytokines and chemokines [9]. Previous studies have confirmed the antiviral immune response of TECs against viral respiratory infections in different species, such as mice and humans [10].

The cell-cell junctions of epithelial cells, including tight and adherens junctions, are vital components of the epithelial barrier. These junction proteins regulate intercellular transport and provide a barrier function against infection [11]. Tight junctions control transport and maintain cellular structure, while adherens junctions regulate cell-cell adhesion and the actin cytoskeleton [12]. AIV infection damages the human apical junction complexes, leading to disruptions in cell permeability [13]. Cell junctions are often targeted by various viruses, such as immunodeficiency virus, hepatitis C virus, and rotavirus in humans [10]. However, the mechanism by which AIV infection damages the epithelial barrier remains unclear.

While methods for the primary culture of TECs have been established in mice and humans, studies in chickens have primarily focused on understanding the immediate host response to viral infections [14]. To address the gaps in understanding the antiviral response mechanism in chicken tracheal epithelium, we aimed to establish a primary culture of airway epithelial cells of chickens. This would provide a valuable resource for experiments in controlled environments, facilitating respiratory disease research. Moreover, polyinosinic-polycytidylic acid (poly [I:C]), functioning as a viral mimic molecule, triggers innate immune responses, prompting the production of antiviral molecules, including pro-inflammatory cytokines and chemokines. Our study sought to establish a method for culturing primary chicken TECs and investigate host antiviral mechanisms in TECs stimulated by HPAIV and poly (I:C).

## MATERIALS AND METHODS

All experiments and animal care protocols were certified by the Ministry of Agriculture and Rural Development of Vietnam (TCVN 8402:2010/TCVN 8400-26:2014).

### Isolation of primary chicken tracheal epithelial cells

Eighteen-day-old embryonic chicken eggs were used to culture the primary chicken TECs. The tracheae from the embryos were isolated, collected in 1× phosphate-buffered saline (PBS), and rinsed once with 1× PBS. The isolated tracheae were dissected into 1-cm sections after removing the surrounding muscle, connective tissue, and mucus. The trachea sections were incubated at 37°C for 2 h in culture medium containing collagenase type 1 (1 mg/mL; Gibco, Grand Island, NY, USA). To halt the enzymatic reaction, the tracheae were gently rinsed and dispersed in the culture media. The epithelial cell sheet was separated from the inner layer of the trachea and placed in a 15-mL tube. Cell pellets were obtained by centrifugation at 1,000×g for 5 min, and the cell pellet was resuspended in 0.1% trypsin-ethylenediaminetetraacetic acid for 5 min to dissociate into single cells. After preculture for 3 to 4 h in a cell culture dish (90×20 mm, #20100; SPL Life Sciences, Pocheon, Korea), the supernatant was collected and cultured by seeding 1.5 ×10^6^ cells in a 12-well plate (#13485; SPL Life Sciences, Korea). The collected cells were then cultured in a culture medium containing 1% L-glutamine (200 mM; Sigma-Aldrich, Saint Louis, MO, USA), 1% non-essential amino acids, 0.1 mM β-mercaptoethanol, 1% penicillin/streptomycin in Dulbecco’s modified essential medium (DMEM)/F-12 (Gibco, USA), and 10% fetal bovine serum (FBS; Gibco, USA). Before use in cell cultures, the plates were coated with 0.1% gelatin at 37°C for 20 min.

### Chicken fibroblast cell line culture

The DF-1 chicken fibroblast cell line (ATCC CRL-12203, USA) was obtained from the American Tissue Culture Collection. DF-1 cells were cultured in Roswell Park Memorial Institute (RPMI) 1640 Complete Medium (Thermo Fisher Scientific, Waltham, MA, USA) containing penicillin (100 IU/mL), streptomycin (100 mg/mL), and 10% FBS (Gibco, USA) in 5% CO_2_ at 37°C in a humidified incubator. This cell line served as the control in this study.

### Reverse transcription-polymerase chain reaction

To confirm the characteristics of primary cultured chicken TECs, reverse transcription-polymerase chain reaction (RT-PCR) analysis of epithelial cell-specific gene makers was performed. Primers designed using NCBI Primer-BLAST ( https://www.ncbi.nlm.nih.gov/tools/primer-blast/, Table 1) were used to confirm the expression of the epithelial cell-specific gene markers, namely retinoic acid responder, fibroblast growth factor (FGF)-binding protein, and virus-activating protease (VAP). The DF-1 cell line was used as the control and glyceraldehyde 3-phosphate dehydrogenase (GAPDH), as the housekeeping gene. Total RNA was extracted from chicken TECs, cDNA was synthesized, and RT-PCR was performed. The PCR product was electrophoresed on 2% agarose gel, stained using DyneStaningSTAR (Dyne Bio INC, Seongnam, Korea), and observed using a UV image analyzer.

### Immunocytochemistry

Immunocytochemistry (ICC) analysis was performed to confirm the morphology and growth pattern of chicken TECs. To characterize epithelial cells, the expression of E-cadherin and zonula occludens (ZO)-1 in the epithelial cell membrane was confirmed. Chicken TECs were cultured in 24-well plates at 37°C. The cells were washed using 1× PBS, fixed for 10 min with 4% paraformaldehyde in 1× PBS, and permeabilized with 1× PBS containing 0.25% Triton X-100. Protein staining was performed using rabbit E-cadherin primary antibody (catalog# 139490, 1:100 dilution; US Biological, Salem, MA, USA) or rabbit ZO-1 polyclonal primary antibody (catalog# 61-7300, 1:50 dilution; Thermo Fisher Scientific, USA) followed by an Alexa Fluor 488 goat anti-rabbit immunoglobulin G (H+L) secondary antibody (catalog# A-11034, 1:1,000 dilution; Thermo Fisher Scientific, USA) incubation. After incubation with antibodies, the cells were rinsed and mounted with Fixed Cell ReadyProbes Reagent (DAPI, blue; Thermo Fisher Scientific, USA). Images were captured using the EVOS FLoid Cell Imaging Station (Life Technologies, Carlsbad, CA, USA).

### Highly pathogenic avian influenza virus infection of tracheal epithelial cells

The HPAIV A/duck/Vietnam/QB1207/2012 (H5N1), which we have previously characterized against several tissues [15,16], was used in this study.

After culturing the TECs, the medium was removed, and the cells were washed with 1× PBS. For virus infection, fresh culture medium containing the H5N1 AIV subtype at a multiplicity of infection (MOI) of 0.1 plaque-forming units (PFU)/cell was added. The cells were then incubated with the virus at 37°C for different durations (0, 3, 6, and 12 h), washed with 1× PBS, and harvested for further study.

### Polyinosinic-polycytidylic acid (poly (I:C)) treatment

Cultured primary TECs were rinsed twice with 1× PBS and treated with poly (I:C) (10 μM; #P1530, Sigma-Aldrich, USA) in the culture medium. The cells were then washed twice using 1× PBS and collected after 12h for gene expression validation. Specifically, the TECs were treated with various concentrations (0, 1, 5, and 10 μg/mL) of poly (I:C) for different durations (0, 12, and 24 h) to select an appropriate final concentration.

### Inhibition of NF-κB and MAPK signaling pathways in tracheal epithelial cells

The cultured TECs were pretreated with BAY 11–7085 (5 μM, 3 h; Sigma-Aldrich, USA), SB202190 (10 μM, 1 h; InvivoGen, San Diego, CA, USA) and SP600125 (25 μM, 1 h; InvivoGen, USA) to inhibit the NF-κB and MAPK signaling pathways, respectively. The cells were then treated with poly (I:C) for 12 h and washed with 1× PBS as described above.

### Immune-related gene expression analysis by reverse transcription-quantitative polymersase chain reaction

Cultured TECs were treated with H5N1 (MOI of 0.1 PFU/cell) or poly (I:C) and incubated at 37°C with 5% CO_2_. The TECs were then collected, and RNA was extracted using TRIzol reagent (Thermo Fisher Scientific, USA) by following the manufacturer’s instructions. For cDNA synthesis, 2 μg of total RNA was treated with 1.0 U DNase I (RNase-free) and used for cDNA synthesis with a RevertAid First Strand cDNA Synthesis Kit (Invitrogen, Carlsbad, CA, USA). The resulting cDNA was diluted to 100 ng/μL using nuclease-free water.

For the reverse transcription-quantitative polymersase chain reaction (RT-qPCR) analysis, Dyne qPCR 2x PreMIX (SYBR Green with low ROX; Dyne Bio INC, Korea) was used in a CFX connect Real-time PCR Detection System (Bio-Rad, Hercules, CA, USA) according to the manufacturer’s protocol. Primers designed with NCBI Primer-BLAST ( https://www.ncbi.nlm.nih.gov/tools/primer-blast/, Table 2) were utilized. The reaction mixture (20 μL) comprised 10 μL of 2× SYBR Green Master Mix, 1 μL each of 10 pM forward and reverse primers, 1 μL of template cDNA, and 7.0 μL of nuclease-free water. The amplification steps were as follows: 5 min at 95°C, 40 cycles of 30 s at 94°C, 50°C to 65°C annealing for 30 s, and extension for 30 s at 72°C. All the experiments were performed independently in triplicate. mRNA expression levels were normalized to those of GAPDH.

### Western blotting

Primary chicken TECs were lysed in 100 μL radio-immunoprecipitation assay (RIPA) buffer supplemented with phosphatase inhibitor and protease inhibitor cocktails (Thermo Fisher Scientific, USA). The cells were collected from the culture plates and transferred to 1.7-mL Eppendorf tubes. The cells were centrifuged at 12,000×g for 15 min at 4°C, and the supernatant was stored at −80°C. Rabbit E-cadherin primary antibody (catalog# 139490, 1:500 dilution; US Biological, USA) and rabbit ZO-1 polyclonal primary antibody (catalog# 61-7300, 1:200 dilution; Thermo Fisher Scientific, USA) were used. Horseradish peroxidase (HRP)-conjugated anti-rabbit or anti-mouse (Thermo Fisher Scientific, USA) secondary antibodies were used based on the primary antibodies. Protein bands were detected using the Western Lightning Plus-ECL substrate (Thermo Fisher Scientific, USA). Protein expression levels were normalized to those of GAPDH (AM4300; Thermo Fisher Scientific, USA).

### Statistical analysis

All data were analyzed using IBM SPSS Statistics for Windows, version 26.0 (IBM Corp., Armonk, NY, USA). Each RT-qPCR experiment was replicated three times. Statistical comparisons were performed in triplicate for two-group comparisons using the student’s t-test. Pairwise comparisons were performed using one-way analysis of variance (ANOVA) followed by Duncan’s post-hoc test. The data were presented as means± standard error of the mean. A difference was considered statistically significant when p<0.05, p<0.01, and p<0.001.

## RESULTS

### Primary chicken tracheal epithelial cell culture

**Figure 1:**
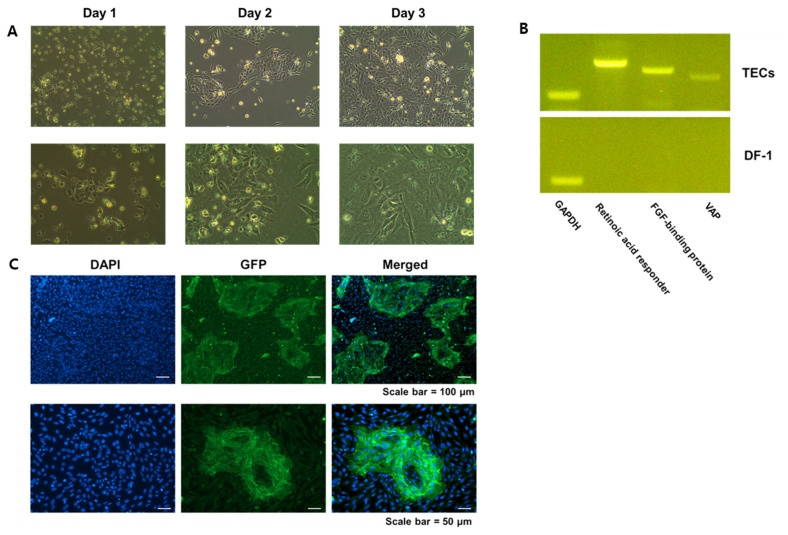
Identification and characterization of primary chicken tracheal epithelial cells (TECs). (A) Morphology and cell growth pattern of TECs 1, 2, and 3 days after primary cell culture observed under a microscope at 100× and 200× magnification after cell attachment; (B) Epithelial cell marker expression of TECs and the DF-1 cell line determined by reverse transcription-polymerase chain reaction (RT-PCR) with mRNA levels of TECs measured 3 days after primary culture compared with those of DF-1 cells; (C) Immunocytochemistry analysis of primary TECs demonstrating expression of epithelial cell surface makers detected using rabbit anti-E-cadherin primary antibody followed by an Alexa Fluor 488 goat anti-rabbit immunoglobulin G (H+L) secondary antibody. Scale bars = 50 μm and 100 μm as indicated.

Chicken TECs were successfully isolated from 18-day-old embryonic chickens. After cell attachment, primary TECs at different incubation periods were observed under the microscope at 100× and 200× (Figure 1A) magnification. The TECs grew as a monolayer of cells with chicken embryonic fibroblasts. Moreover, these cells showed polygonal cell morphology, which is characteristic of epithelial cells. The cultured primary TECs formed colony-like cell clusters and proliferated around the clusters. In this study, 2 to 3 days were required for cells to attach and grow to 70% to 80% confluence or more in primary culture.

### Identification of tracheal epithelial cells

Epithelial cell characteristics were observed in TECs by RT-PCR and ICC analyses. The characteristics of primary TECs were confirmed using epithelial cell gene markers, including retinoic acid responder, FGF-binding protein, and VAP. The expression of epithelial cell-specific marker genes was detected in the TECs but not in the DF-1 cells (Figure 1B). In contrast, GAPDH expression did not differ between the chicken TECs, and the DF-1 cell line (Figure 1B).

To further confirm the morphology and growth pattern of the TECs, ICC analysis was performed, focusing on the expression of an epithelial membrane marker (Figure 1C). As expected, the membrane marker, E-cadherin was detected in the cultured primary TECs but not in the chicken fibroblast DF-1 cell line. The growth patterns showed the same colony-type cell clusters as described above (Figure 1). These results indicate that epithelial cells isolated from the trachea have properties based on the expression of several known epithelial-specific marker genes and junction proteins in the epithelial cell membrane.

### Antiviral response of tracheal epithelial cells to HPAIV H5N1 subtype

**Figure 2:**
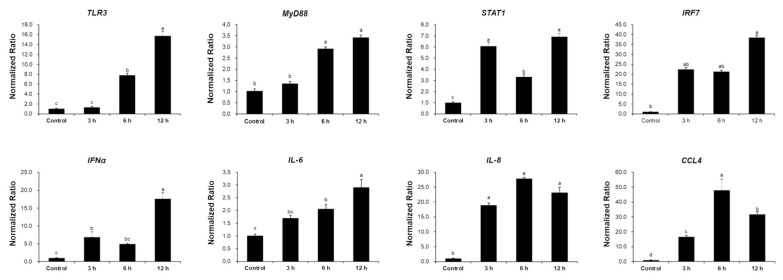
Antiviral gene expression in primary chicken tracheal epithelial cells (TECs). H5N1 (multiplicity of infection [MOI] of 0.1 plaque-forming units [PFU]/cell) infection increases the expression of immune-related genes (*TLR3*, *MYD88*, *STAT1*, *IRF7*, *IFN-α*, *IL-6*, *IL-8*, and *CCL4*) relative to that of uninfected control cells. Gene expression is expressed as fold-change relative to the control. Statistical analysis was performed with pairwise comparisons followed by Duncan’s post-hoc tests. ^a–d^ Different letters indicate significant differences at p<0.05. *TLR*, toll-like receptor; *MYD*, myeloid differentiation primary response; *STAT*, signal transducers and activators of transcription; *IRF*, interferon regulatory factor; *IFN*, interferon; *IL*, interleukin; *CCL*, chemokine (C-C motif) ligand.

To investigate the cellular immune response to HPAIV infection, the TECs were infected with H5N1 virus (MOI of 0.1 PFU/cell) in the culture media. After H5N1 infection, the TECs were collected to validate gene expression using RT-qPCR (Figure 2). The evaluation focused on measuring mRNA expression of antiviral genes in response to HPAIV infection and showed a significant increase in antiviral gene expression in the TECs infected with the H5N1 subtype (Figure 2). Over time, TLR3 and myeloid differentiation primary response (MYD) 88 expression increased and reached a peak value of 15.74- and 3.42-fold, respectively at 12 h. STAT1 expression significantly increased by 6.08-fold, 3.5-fold, and 6.92-fold, respectively, at all times of culture. Interferon (IFN)-α and interferon regulatory factor (IRF) 7 expression peaked at 12 h of incubation by 17.57- and 39.44-fold, respectively. Chemokine (C-C motif) ligand (CCL) 4 and interleukin (IL)-8 expression peaked at 6 h of incubation by 47.98- and 27.85-fold, respectively. Moreover, IL-6 expression increased over time and peaked at 12 h by 2.91-fold. These data indicate that primary chicken TECs infected with HPAIV recognize pathogens, trigger antiviral response, and produce antiviral molecules. Thus, TECs can express several innate immune genes as well as pro-inflammatory cytokines and chemokines in response to pathogens, including AIV.

### Expression of antiviral genes stimulated by poly (I:C) treatment in tracheal epithelial cells

**Figure 3:**
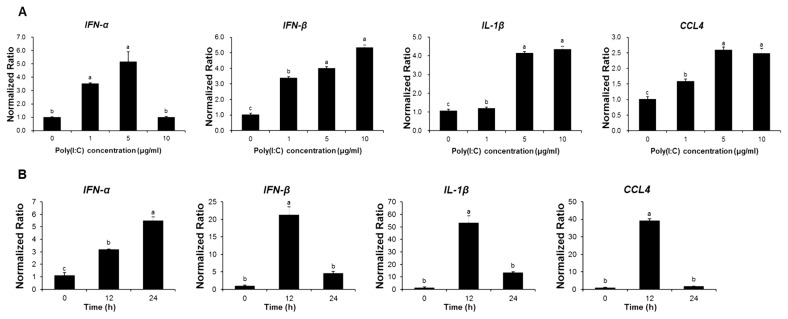
Effects of polyinosinic-polycytidylic acid (poly [I:C]) treatment on cultured chicken TECs as determined by mRNA expression patterns. Poly (I:C) treatment increases the expression of antiviral genes (*IFNs*, *IL-1β*, and *CCL4*) relative to that of untreated control cells. Gene expression in tracheal epithelial cells (TECs) treated with different (A) poly (I:C) concentrations; or (B) treatment times compared with that of the control. Statistical analysis was performed using pairwise comparisons followed by Duncan’s post-hoc tests. ^a–c^ Different letters indicate significant differences at p<0.05. *IFN*, interferon; *IL*, interleukin; *CCL*, chemokine (C-C motif) ligand.

To investigate the impact of poly (I:C) on antiviral gene expression, the TECs were treated with various concentrations of poly (I:C). Except for the expression of IFN-α, that of antiviral genes and chemokines, such IFN-β, IL-1β, and CCL4 increased in a concentration-dependent manner (Figure 3A). Additionally, IFN-β, IL-1β, and CCL4 reached their peak expression levels 12 h after treatment with 10 μg/mL poly (I:C) (Figure 3B).

**Figure 4:**
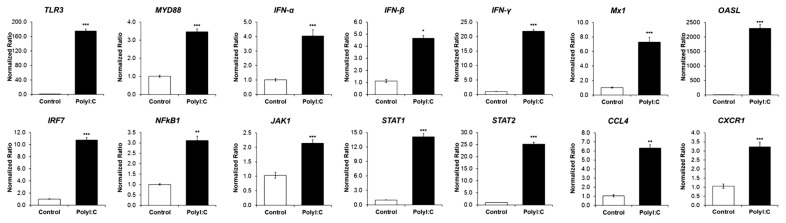
Gene expression of immune responses in tracheal epithelial cells (TECs) after polyinosinic-polycytidylic acid (poly [I:C]) treatment. Gene expression measured after poly (I:C) (10 μg/mL) treatment for 12 h using reverse transcription-quantitative polymerase chain reaction (RT-qPCR) compared with that of *GAPDH*. Gene expression was expressed as fold-change relative to that of the control. Significance was evaluated using Student’s t-test at * p<0.05, ** p<0.01, and *** p<0.001 (compared with the control group). *GAPDH*, glyceraldehyde 3-phosphate dehydrogenase.

We also measured the expression of genes related to the TLR and JAK/STAT signaling pathways as well as that of pro-inflammatory cytokines and chemokines in TECs 12 h after treatment. Compared with the control cells, the TECs treated with poly (I:C) demonstrated TLR3 upregulation. TECs treated with poly (I:C) significantly induced the expression of transcription factors, IRF7 and NF-κB1 by 10.79- and 3.12-fold, respectively. Moreover, MYD88, the signaling adaptor for TLRs (except TLR3), was upregulated by 3.45-fold (Figure 4).

Treatment of the TECs with poly (I:C) on TECs highly induced the expression of IFN-α, IFN-β, and IFN-γ by 4.06-, 4.66-, and 21.85-fold, respectively. The expressions of JAK1, STAT1, and STAT2 were significantly upregulated by 2.02-, 14.09-, and 25.16-fold, respectively (Figure 4). Moreover, MX1 and 2′-5′-oligoadenylate synthetase-like protein (OASL) were significantly upregulated by 7.29-fold, and 2300-fold, respectively. Poly (I:C) treatment also caused upregulation of CCL4 and C-X-C chemokine receptor (CXC) R1 expression by 6.33- and 3.23-fold, respectively (Figure 4).

### Regulation of immune networks through the NF-κB and MAPK signaling pathways in poly (I:C)-stimulated tracheal epithelial cells

**Figure 5:**
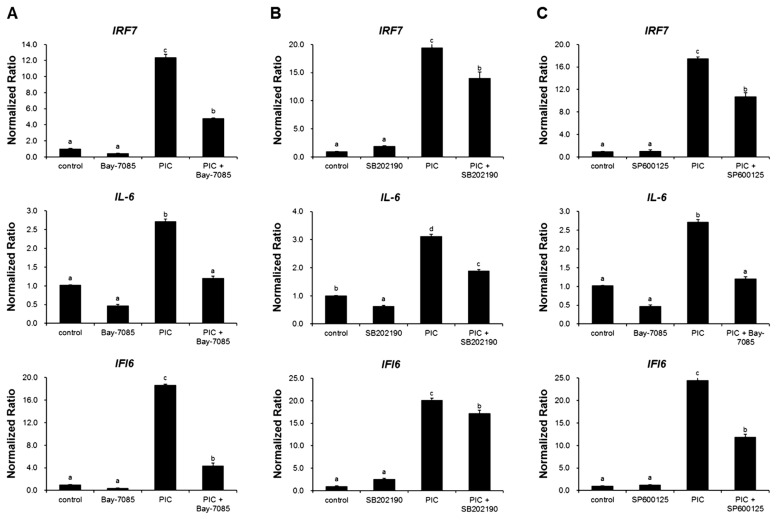
Assessment of antiviral effects of signaling pathways on tracheal epithelial cells (TECs) using pathway inhibitors. TECs treated with (A) NF-κB inhibitor (BAY 11–7085); (B) p38 MAPK inhibitor (SB202190); and (C) JNK MAPK inhibitor (SP600125) before treatment with polyinosinic-polycytidylic acid (poly [I:C]). Each pathway inhibitor blocked the poly (I:C)-stimulated antiviral gene expression. After treatment with poly (I:C), the cells were harvested for total RNA extraction. mRNA levels were determined using RT-qPCR. ^a–d^ Different letters indicate significant differences at p<0.05. NF-κB, nuclear factor kappa B; JNK, c-Jun NH2-terminal kinase; MAPK, mitogen-activated protein kinase.

To validate the impact of the NF-κB and MAPK signaling pathways in TECs, pathway inhibitors were employed to suppress their activation. Compared with the control TECs, the ones treated with poly (I:C) exhibited significantly increased antiviral response, leading to a significantly high expression of IRF7, IL-6, and IFN-α inducible protein 6 (IFI6) (Figure 5).

Furthermore, the results demonstrated that treatment with the signaling pathway inhibitors, namely BAY 11–7085 (NF-κB inhibitor), SB202190 (p38 MAPK inhibitor), and SP600125 (c-Jun NH2-terminal kinase [JNK] MAPK inhibitor) effectively decreased the antiviral effects induced by poly (I:C) treatment. The expression of the *IRF7*, *IL-6*, and *IFI6* genes was significantly lesser in the TECs treated with each signaling pathway inhibitor and poly (I:C) than that of those in the cells treated only with poly (I:C) (Figure 5).

### Effects of poly (I:C) treatment on junction proteins in tracheal epithelial cells

**Figure 6:**
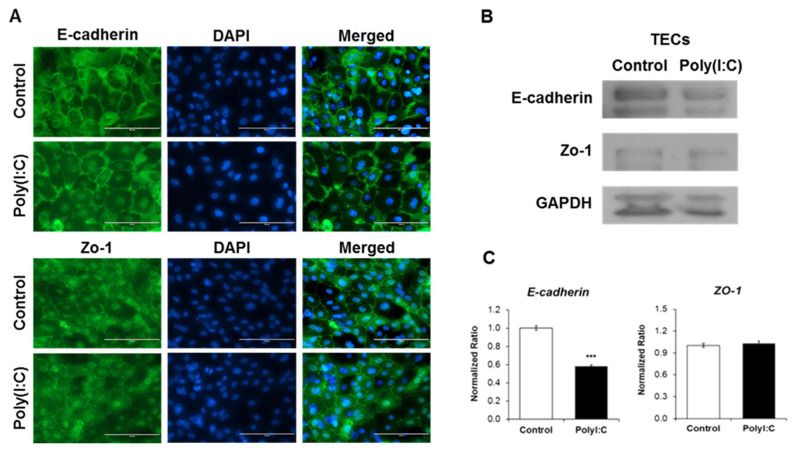
Visualization of expression of epithelial cell junction protein after polyinosinic-polycytidylic acid (poly [I:C]) treatment in tracheal epithelial cells (TECs). Junction proteins of TECs were analyzed 24 h after treatment in the control and poly (I:C) treatment groups by (A) immunocytochemistry (ICC) analysis; and (B) western blotting. (C) mRNA levels of E-cadherin decreased with poly (I:C) treatment after 24 h relative to those of negative control cells. The mRNA levels of ZO-1 showed no significant change. mRNA expression levels were normalized to those of GAPDH. *** p<0.001 compared with the control group. ZO, zonula occludens; GAPDH, glyceraldehyde 3-phosphate.

The impact of poly (I:C) treatment on junction proteins in the TECs, which regulate the physical barrier function, was investigated. After 24 h of poly (I:C) stimulation, ICC and western blotting were performed to assess the expression patterns of junction proteins, specifically E-cadherin and ZO-1. ICC analysis revealed no significant difference between the control and poly (I:C)-treated TECs (Figure 6A). However, western blotting results demonstrated that poly (I:C) treatment led to decreased protein expression levels of E-cadherin in adherens junctions, while those of ZO-1 in tight junctions did not change significantly (Figure 6B). Similarly, the mRNA expression of E-cadherin decreased in the TECs after poly (I:C) treatment, whereas that of ZO-1 did not differ significantly (Figure 6C). The results indicate that TECs act as a physical barrier and poly (I:C) treatment results in the loss of epithelial cell junction proteins.

## DISCUSSION

The respiratory epithelium, which is crucial for defense against respiratory infections, is a primary target of influenza viruses in humans and mammals. While the defense mechanisms of human respiratory epithelial cells are well documented, those of chicken TECs against avian respiratory diseases are unclear [17]. This study demonstrated that treatment of TECs with H5N1 virus or poly (I:C) induced an immune response by the activation of signaling pathways and production of antiviral molecules while weakening the physical barrier between TECs.

When AIVs are recognized by host PRRs, including TLRs, through adaptor receptors, such as MYD88, NF-κB, and IRF7 are activated to regulate the expression levels of cytokines, chemokines, and interferon-stimulated genes (ISGs) [18]. Type I and type II IFNs trigger ISGs through the JAK/STAT signaling pathway [19]. In the present study, after H5N1 infection of TECs, the expression level of TLR3, MYD88, IRF7, IFN-α, and IL-6 peaked at 12 h. The expression level of IL-8 and CCL4 peaked at 6 h, while that of STAT1 peaked at 3 and 12 h. The decreased STAT1 expression may be attributed to the blocking of IFN-induced signals through inhibition of the IFN receptor by the AIVs [19]. These results are similar to those of previous *in vivo* studies on the trachea [8,15,20]. Our results indicate that TECs induce innate and adaptive immune responses and produce antiviral molecules against H5N1 infection.

Poly (I:C), a dsRNA molecule recognized by TLR3, in creases host immunity against AIV and induces antiviral molecules production [14]. TECs were treated with poly (I:C) to investigate whether they could serve as an *in vitro* model for respiratory diseases, such as AIV infection. Our data indicate that treatment of TECs with poly (I:C) increased the expression of antiviral molecules, including IFN-α, IFN-β, IL-1β, and CCL4. Therefore, poly (I:C) treatment induces gene expression patterns similar to that due to H5N1 infection in *in vitro* experiments.

Additionally, the expression of TLR3, MYD88, IRF7, and NF-κB was found to have increased after treatment of TECs with poly (I:C). The IFN molecules that were produced activated the JAK/STAT signaling pathway, leading to the expression of ISGs, such as Mx1 and OASL, as well as inflammatory cytokines and chemokines, like CCL4 and CXCR1. The chicken *Mx1* gene, regulated by type I IFNs, acts as an antiviral restriction factor against influenza viruses [21]. Moreover, OASL, which is an antiviral protein, inhibits viral replication and expression in response to several viruses [22]. These results indicate that TECs induce an antiviral response after poly (I:C) treatment and thus play a role in host defense.

Previous studies have revealed that the NF-κB and MAPK signaling pathways are important for inflammatory responses [23]. They are involved in AIV-triggered inflammatory response and mediate the production of pro-inflammatory cytokines and chemokines stimulated with AIV [24]. Moreover, the H5N1 virus induces autophagy-mediated inflammatory responses by activation of the NF-κB and p38 MAPK signaling pathways [25]. IL-6 is involved in overall immune response and protects cells from apoptosis due to an interaction between NF-κB and p38 MAPK [26]. IFI6 is an ISG belonging to the FAM14 protein family. The expression of IFI6 and IRF7 increases in response to HPAIV and is regulated by the NF-κB and MAPK signaling pathways. The expression of IFI6 and IRF7 increases in response to HPAIV and is regulated by the NF-κB and MAPK signaling pathways [27]. In the present study, treatment with NF-κB and MAPK signaling pathway inhibitors efficiently prevented the poly (I:C)-mediated immune response in TECs. These results imply that the NF-κB and MAPK signaling pathways may contribute to the antiviral inflammatory response against AIV infection.

Epithelial barrier function is influenced by the barrier integrity of epithelial cells and apical junction complexes, including tight and adherens junctions [28]. Our results showed that poly (I:C) treatment significantly decreased both mRNA and protein expression of E-cadherin in adherens junctions. However, while the protein expression of ZO-1, which is a tight junction component, decreased, its mRNA expression did not show a significant difference. Epithelium protects the host from pathogens through barriers formed by intracellular junctions. H5N1 viruses downregulate junction protein expression in respiratory epithelial cell lines of several species and the lungs of H5N1 virus-infected mice [29]. Moreover, activation of TLR3 disrupts the corneal epithelial barrier in humans [30]. In contrast, tight junctions (ZO-1) are maintained despite AIV infection in primary porcine airway epithelial cells and a human epithelial cell line [7]. It appears that differentiated cells can revert to specialized basal cells with barrier function in the tracheal epithelium [31]. Other studies have confirmed that AIV infection damages intracellular junctions and junction proteins [13]. Furthermore, poly (I:C) can damage the respiratory physical barrier by downregulating intracellular junction proteins in epithelial cells [32]. Our results demonstrated that chicken TECs act as a physical barrier against viral infection.

Chicken TECs are the initial defense against external pathogens and function as a physical barrier, making them a suitable model for investigating respiratory diseases. This study demonstrated that poly (I:C)-treated TECs exhibit pathway activation, molecule production, and cellular damage similar to AIV-infected cells. Chicken TEC cultures can serve as a valuable *in vitro* model for studying avian respiratory diseases. While these findings contribute to understanding antiviral mechanisms in chickens, further studies are required to explore the intricate interactions with other immune-related factors and enhance our understanding of tracheal physiology.

## Figures and Tables

**Table 1:** Primer sequences used for reverse transcription-polymerase chain reaction analysis

Gene	Primer	Nucleotide sequences (5′ → 3′)	GenBank Accession No.
Retinoic acid responder	F	ACATCAACTCCCACGAGGCGTCC	NM_204534.5
	R	ACTGCTGCCAACAATGGCCAAGC	
FGF-binding protein	F	TGGAGTTTGGACACCTCCGGCT	XM_040671804.2
	R	TGCAGCACTCATGTCGGTCACC	
*VAP*	F	TAGTGGCGTGGCTCTCAAAC	NM_205022.2
	R	AAGACACCAATGAGCAGGCA	
*GAPDH*	F	TGCTGCCCAGAACATCATCC	NM_204305
	R	ACGGCAGGTCAGGTCAACAA	

FGF, fibroblast growth factor; *VAP*, virus-activating protease; *GAPDH*, glyceraldehyde 3-phosphate dehydrogenase.

**Table 2:** Primer sequences used for reverse transcription-quantitative polymerase chain reaction analysis

Gene	Primer	Nucleotide sequences (5′ → 3′)	GenBank accession No.
*IFN-α*	F	GAGCAATGCTTGGACAGCAG	GU119896.1
	R	GAGGTTGTGGATGTGCAGGA	
*IFN-β*	F	CTTGCCCACAACAAGACGTG	NM_001024836.1
	R	TGTTTTGGAGTGTGTGGGCT	
*IL-1β*	F	TGCCTGCAGAAGAAGCCTCG	NM_204524.1
	R	CTCCGCAGCAGTTTGGTCAT	
*CCL4*	F	CTTCACCTACATCTCCCGGC	NM_001030360.2
	R	CTGTACCCAGTCGTTCTCGG	
*TLR3*	F	GGTCCAGCTTTCAAGAGCCT	MF576162.1
	R	GCAACACCAGAGTACCGTGA	
*MYD88*	F	TTGTAACTTCGCTATTGGTATTCC	NM_001030962.4
	R	TTCCGTGATGTGTCTTCCTTC	
*IRF7*	F	CTCTCCCCTCCTCCAAAAGC	NM_205372
	R	AGCGAAGGAGGAATGAACCC	
*NFκB1*	F	AGAAAAGCTGGGTCTTGGCA	NM_205134
	R	CCATCTGTGTCAAAGCAGCG	
*IFN-γ*	F	AACAACCTTCCTGATGGCGT	NM_205149.1
	R	TGAAGAGTTCATTCGCGGCT	
*JAK1*	F	TTCTGGGAGCTTAAAGGAGTATCT	NM_204870
	R	CTGACGGGAGCCCAAGTAGT	
*STAT1*	F	TTGTAACTTCGCTATTGGTATTCC	NM_001012914
	R	TTCCGTGATGTGTCTTCCTTC	
*STAT2*	F	ATATTTTCACTGGGGCTGGTA	NM_001030626
	R	TGGGGATCACGAAACATAAA	
*MX1*	F	AGCCATAGAACAAGCCAGAA	NM_204609.1
	R	GGTACTGGTAAGGAAGGTGG	
*OASL*	F	TCAAGACCGTCAAGGGCG	NM_001397447.1
	R	GGACTGGTGATGCTGACTCC	
*CXCR1*	F	TTACGCTGACGAACTCTTGG	NM_001282432.1
	R	TTCATTACGGCATGGGGAAG	
*IL-6*	F	GCAGGACGAGATGTGCAAGA	NM_204628.1
	R	ATTTCTCCTCGTCGAAGCCG	
*IL-8*	F	GGCTTGCTAGGGGAAATGA	NM_205498.1
	R	AGCTGACTCTGACTAGGAAACTGT	
*IFI6*	F	AGGCAAAATCCTTTGGGGGA	NM_001001296.6
	R	AAGAGGTGGCCTCATTGGAC	
*E-cadherin*	F	GACAGGGACATGAGGCAGAA	XM_046925643.1
	R	GCCGTGACAATGCCATTCTC	
*ZO-1*	F	GCCTGAATCAAACCCAGCAA	XM_046925214.1
	R	TATGCGGCGGTAAGGATGAT	
*GAPDH*	F	TGCTGCCCAGAACATCATCC	NM_204305
	R	ACGGCAGGTCAGGTCAACAA	

*IFN*, interferon; *IL*, interleukin; *CCL*, chemokine (C-C motif) ligand; *TLR*, toll-like receptor; *MYD*, myeloid differentiation primary response; *IRF*, interferon regulatory factor; *NFκB*, Nuclear factor kappa B; *JAK*, Janus kinase; *STAT*, signal transducers and activators of transcription; *MX1*, MX dynamin like GTPase 1; *OASL*, 2′-5′-oligoadenylate synthetase-like protein; *CXCR*, C-X-C chemokine receptor; *IFI*, interferon regulatory factor; *ZO*, zonula occludens; *GAPDH*, glyceraldehyde 3-phosphate dehydrogenase.

## References

[1] Alexander DJ (2007). An overview of the epidemiology of avian influenza. Vaccine.

[2] Chan PKS (2002). Outbreak of avian influenza A(H5N1) virus infection in Hong Kong in 1997. Clin Infect Dis.

[3] Swayne DE (2007). Understanding the complex pathobiology of high pathogenicity avian influenza viruses in birds. Avian Dis.

[4] Ibricevic A, Pekosz A, Walter MJ (2006). Influenza virus receptor specificity and cell tropism in mouse and human airway epithelial cells. J Virol.

[5] Iwasaki A, Foxman EF, Molony RD (2017). Early local immune defences in the respiratory tract. Nat Rev Immunol.

[6] Kato A, Schleimer RP (2007). Beyond inflammation: airway epithelial cells are at the interface of innate and adaptive immunity. Curr Opin Immunol.

[7] Wu NH, Yang W, Beineke A (2016). The differentiated airway epithelium infected by influenza viruses maintains the barrier function despite a dramatic loss of ciliated cells. Sci Rep.

[8] Vu TH, Hong Y, Truong AD (2022). The highly pathogenic H5N1 avian influenza virus induces the mitogen-activated protein kinase signaling pathway in the trachea of two Ri chicken lines. Anim Biosci.

[9] Kato A, Schleimer RP (2007). Beyond inflammation: airway epithelial cells are at the interface of innate and adaptive immunity. Curr Opin Immunol.

[10] Daidoji T, Kajikawa J, Arai Y, Watanabe Y, Hirose R, Nakaya T (2020). Infection of human tracheal epithelial cells by H5 avian influenza virus is regulated by the acid stability of hemagglutinin and the pH of target cell endosomes. Viruses.

[11] Anderson JM (2001). Molecular structure of tight junctions and their role in epithelial transport. Physiology.

[12] Linfield DT, Raduka A, Aghapour M, Rezaee F (2021). Airway tight junctions as targets of viral infections. Tissue Barriers.

[13] Short KR, Kasper J, van der Aa S (2016). Influenza virus damages the alveolar barrier by disrupting epithelial cell tight junctions. Eur Respir J.

[14] Barjesteh N, O’Dowd K, Vahedi SM (2020). Antiviral responses against chicken respiratory infections: Focus on avian influenza virus and infectious bronchitis virus. Cytokine.

[15] Lee J, Hong Y, Vu TH (2022). Influenza A pathway analysis of highly pathogenic avian influenza virus (H5N1) infection in genetically disparate Ri chicken lines. Vet Immunol Immunopathol.

[16] Vu TH, Hong Y, Truong AD (2022). Cytokine-cytokine receptor interactions in the highly pathogenic avian influenza H5N1 virus-infected lungs of genetically disparate Ri chicken lines. Anim Biosci.

[17] Esnault E, Bonsergent C, Larcher T (2011). A novel chicken lung epithelial cell line: characterization and response to low pathogenicity avian influenza virus. Virus Res.

[18] Barjesteh N, Taha-Abdelaziz K, Kulkarni RR, Sharif S (2019). Innate antiviral responses are induced by TLR3 and TLR4 ligands in chicken tracheal epithelial cells: communication between epithelial cells and macrophages. Virology (Lond).

[19] Fleming SB (2016). Viral inhibition of the IFN-induced JAK/STAT signalling pathway: development of live attenuated vaccines by mutation of viral-encoded IFN-antagonists. Vaccines (Basel).

[20] Vu TH, Heo J, Hong Y (2023). HPAI-resistant Ri chickens exhibit elevated antiviral immune-related gene expression. J Vet Sci.

[21] Haller O, Staeheli P, Schwemmle M, Kochs G (2015). Mx GTPases: dynamin-like antiviral machines of innate immunity. Trends Microbiol.

[22] Melchjorsen J, Kristiansen H, Christiansen R (2009). Differential regulation of the OASL and OAS1 genes in response to viral infections. J Interferon Cytokine Res.

[23] Kaminska B (2005). MAPK signalling pathways as molecular targets for anti-inflammatory therapy—from molecular mechanisms to therapeutic benefits. Biochim Biophys Acta Proteins Proteom.

[24] Xing Z, Cardona CJ, Anunciacion J, Adams S, Dao N (2010). Roles of the ERK MAPK in the regulation of proinflammatory and apoptotic responses in chicken macrophages infected with H9N2 avian influenza virus. J Gen Virol.

[25] Pan H, Zhang Y, Luo Z (2014). Autophagy mediates avian influenza H5N1 pseudotyped particle-induced lung inflammation through NF-kappaB and p38 MAPK signaling pathways. Am J Physiol Lung Cell Mol Physiol.

[26] Craig R, Larkin A, Mingo AM (2000). p38 MAPK and NF-κB collaborate to induce interleukin-6 gene expression and release: evidence for a cytoprotective autocrine signaling pathway in a cardiac myocyte model system. J Biol Chem.

[27] Park JW, Ndimukaga M, So J (2023). Molecular analysis of chicken interferon-alpha inducible protein 6 gene and transcriptional regulation. J Anim Sci Technol.

[28] Gao N, Rezaee F (2022). Airway epithelial cell junctions as targets for pathogens and antimicrobial therapy Pharmaceutics.

[29] Ruan T, Sun Y, Zhang J (2022). H5N1 infection impairs the alveolar epithelial barrier through intercellular junction proteins via Itch-mediated proteasomal degradation. Commun Biol.

[30] Wei J, Jiang H, Gao H, Wang G (2015). Activation of toll like receptor-3 induces corneal epithelial barrier dysfunction. Biochem Biophys Res Commun.

[31] Tata PR, Mou H, Pardo-Saganta A (2013). Dedifferentiation of committed epithelial cells into stem cells in vivo. Nature.

[32] Rezaee F, Meednu N, Emo JA (2011). Polyinosinic: polycytidylic acid induces protein kinase D–dependent disassembly of apical junctions and barrier dysfunction in airway epithelial cells. J Allergy Clin Immunol.

